# Bagging Statistical Network Inference from Large-Scale Gene Expression Data

**DOI:** 10.1371/journal.pone.0033624

**Published:** 2012-03-30

**Authors:** Ricardo de Matos Simoes, Frank Emmert-Streib

**Affiliations:** Computational Biology and Machine Learning Lab, Center for Cancer Research and Cell Biology, School of Medicine, Dentistry and Biomedical Sciences, Queen's University Belfast, Belfast, United Kingdom; UCLA-DOE Institute for Genomics and Proteomics, United States of America

## Abstract

Modern biology and medicine aim at hunting molecular and cellular causes of biological functions and diseases. Gene regulatory networks (GRN) inferred from gene expression data are considered an important aid for this research by providing a map of molecular interactions. Hence, GRNs have the potential enabling and enhancing basic as well as applied research in the life sciences. In this paper, we introduce a new method called BC3NET for inferring causal gene regulatory networks from large-scale gene expression data. BC3NET is an ensemble method that is based on *bagging* the C3NET algorithm, which means it corresponds to a Bayesian approach with noninformative priors. In this study we demonstrate for a variety of simulated and biological gene expression data from *S. cerevisiae* that BC3NET is an important enhancement over other inference methods that is capable of capturing biochemical interactions from transcription regulation and protein-protein interaction sensibly. An implementation of BC3NET is freely available as an R package from the CRAN repository.

## Introduction

Gene networks represent the *blueprint* of the causal interplay between genes and their products on all molecular levels [1]–[6]. Gene regulatory networks (GRN) inferred from large-scale gene expression data aim to represent signals from these different levels of the gene network. The inference, analysis and interpretation of a GRN is a daunting task due to the fact that the concentrations of mRNAs provide only indirect information about interactions occurring between genes and their gene products (e.g., protein interactions). The reason for this is that DNA microarrays measure only the concentration of mRNAs rather than the binding, e.g., between proteins or between a transcription factor and the DNA. Despite the increased community effort in recent years [7], [8] and a considerable number of suggested inference methods [9]–[19] there is an urgent need to further advance our current methods to provide reliable and efficient procedures for analyzing the increasing amount of data from biological, biomedical and clinical studies [20]–[22]. For this reason, this field is currently vastly expanding. A detailed review for many of the most widely used methods can be found in [15], [18], [23]–[26].

A major problem for the inference of regulatory networks are the intricate characteristics of gene expression data. These data are high-dimensional, in the order of the genome size of the studied organism, and nonlinear due to the intertwined connection of the underlying complex regulatory machinery including the multilevel regulation structures (DNA, mRNA, protein, protein complexes, pathways) and turnover rates of the measured mRNAs, products and proteins. Further, gene expression data for network inference are large-scale, although, the “Large 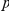 Small 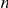
” [27] problem holds, because the number of explanatory variables (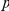 genes) exceeds the number of observations (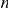 microarray samples). In addition, technical noise and outliers can make it difficult to gain access to the true biological signal of the expression measurement itself.

The main contribution of this paper is to introduce a new network inference method for gene expression data. The principle idea of our method is based on *bootstrap aggregation* [28], [29], briefly called *bagging*, in order to create an ensemble version of the network inference method C3NET [9]. For this reason we call our new method bagging C3NET (BC3NET). The underlying procedure of BC3NET is to generate an ensemble of bootstrap datasets from which an ensemble of networks is inferred by using C3NET. Then the obtained inferred networks are aggregated resulting in the final network. For the last step we employ statistical hypotheses tests removing the need to select a threshold parameter manually. Instead, a significance level with a clear statistical interpretation needs to be selected. This is in contrast with other studies, e.g., [30].

Given the challenging properties of gene expression data, briefly outlined above, BC3NET is designed to target these in the following way. First, BC3NET is based on statistical estimators for mutual information values capable of capturing nonlinearities in the data. Second, in order to cope with noise and outliers in expression data, we employ bagging because it has the desirable ability to reduce the variance of estimates [28]. Computationally, this introduces an additional burden, and a necessary prerequisite for any method to be used in combination with bagging is its tractability to be applicable to a bootstrap ensemble. C3NET is computationally efficient to enable this, even for high-dimensional massive data.

There are a few network inference methods that are similar to BC3NET. The method GENIE3, which was best performer in the DREAM4 *In Silico Multifactorial challenge* [31], employs also an ensemble approach, however, in combination with regression trees, e.g., in form of *Random Forests* [32]. In [30] a bootstrap approach has been used in combination with Bayesian networks to estimate confidence levels for features. However, we want to emphasize that, in contrast to BC3NET, both methods [30], [31] do not provide a statistical procedure for determining an optimal confidence threshold parameter. Finally, we note that also for ARACNe a bootstrap version has been introduced [33], which has so far been used for inferring subnetworks around selected transcription factors [34], [35].

## Methods

### The BC3NET approach for GRN inference

In general, mutual information based gene regulatory network inference methods consists of three major steps. In the first step, a mutual information matrix is obtained based on mutual information estimates for all possible gene pairs in a gene expression data set. In the second step, a hypothesis test is performed for each mutual information value estimate. Finally, in the third step, a gene regulatory network is inferred from the significant mutual information values, according to a method specific procedure.

**Figure 1:**
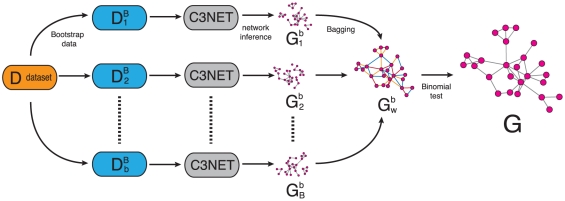
BC3NET algorithm: The gene regulatory network 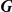
 **is inferred from a bootstrap ensemble generated from a single gene expression dataset** 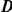 **.** For each generated dataset in the ensemble, 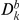, a network, 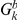, is inferred using C3NET. From 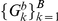 an aggregated network 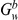 is obtained whose edges are used as test statistics to obtain the final network 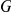.

The basic idea of BC3NET is to generate from one dataset 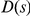, consisting of 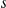 samples, an ensemble of 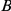 independent bootstrap datasets 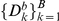 by sampling from 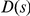 with replacement by using a non-parametric bootstrap [36] with 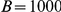. Then, for each generated data set 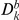 in the ensemble, a network 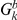 is inferred by using C3NET [9]. From the ensemble of networks 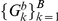 we construct one weighted network
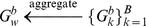
(1)which is used to determine the statistical significance of the connection between gene pairs. This results in the final binary, undirected network 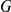. Fig. 1 shows a schematic visualization of this procedure.

A base component of BC3NET is the inference method C3NET introduced in [9], which we present in the following in a modified form to obtain a more efficient implementation. Briefly, C3NET consists of three main steps. First, mutual information values among all gene pairs are estimated. Second, an extremal selection strategy is applied allowing each of the 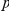 genes in a given dataset to contribute *at most* one edge to the inferred network. That means we need to test only 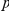 different hypotheses and not 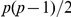. This potential edge corresponds to the hypothesis test that needs to be conducted for each of the 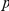 genes. Third, a multiple testing procedure is applied to control the type one error. In the above described context, this results in a network 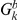.

In order to test the statistical significance of the connection between gene pairs BC3NET utilizes the edge weights of the aggregated network 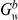 as test statistics. The edge weights of 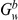 are componentwise defined by
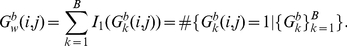
(2)Here 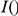 is the indicator function which is 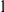 if its argument is 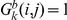 and 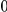 otherwise. This expression corresponds to the number of networks in 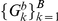 which have an edge between gene 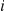 and 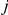. For brevity, we write in the following 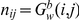. From Eqn. 2 follows that 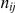 assumes integer values in 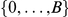. Based on the test statistic 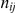, we formulate the following null hypothesis which we test for each gene pair 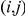.


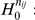
 The number of networks 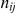 in the ensemble 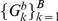 with an edge between gene 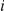 and 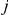 is less than 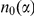.

Here the cut-off value 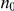 depends on the significance level 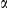. Due to the independence of the bootstrap datasets we assume the null distribution of 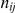 to follow a binomially distributed 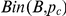, whereas 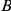 corresponds to the size of the bootstrap ensemble and 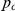 is the probability that two genes are connected by chance. The parameter 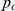 relates to a population of networks, estimated from randomized data by using BC3NET, and corresponds to the fraction of randomly inferred edges in the bootstrap population (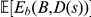) divided by the total number of possible edges in this population (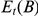) that means
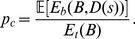
(3)The maximal number of gene pairs that can be formed from 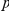 genes in 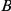 bootstrap datasets is given by
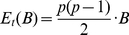
(4)This value is independent of the sample size. 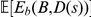 corresponds to the expectation value of the number of randomly inferred edges for a population of an ensemble of bootstrap datasets of size 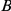. Because 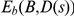 is a random variable it is necessary to average over all possible bootstrap datasets of size 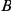 with sample size 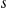. On a theoretical note we remark that these bootstrap datasets constitute a population that specifies a probability mass function (pmf) for which the expectation of 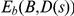 needs to be evaluated. Due to the fact that this pmf is unknown the value of 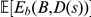 needs to be estimated.

In order to estimate 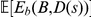 we randomize the data to estimate the number of edges randomly inferred in an bootstrap ensemble of size 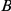, 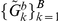


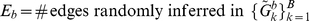
(5)Using 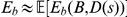 as plug-in estimator for Eqn. 3 we obtain an estimate for 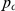. This allows us to calculate a p-value for each gene pair 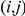 and a given test statistic 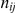, given by Eqn. 2, from the null distribution of 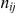 by
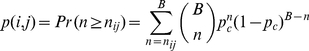
(6)Here 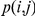 is the probability to observe 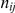 or more edges by chance in a bootstrap ensemble of size 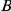 and sample size 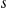.

Because we need to test 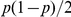 hypotheses simultaneously (one for each gene pair) we need to apply a multiple testing correction (MTC) [37], [38]. For our analysis we are using a Bonferroni procedure for a strong control of the family-wise error rate (FWER). Typically, procedures controlling the FWER are more conservative than procedures controlling, e.g., the false discovery rate (FDR) by making only mild assumptions about the underlying data [39], [40]. Based on these hypotheses tests the final network 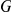 is componentwise defined by
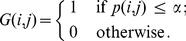
(7)That means if the connection between a gene pair is statistically significant they are connected by and edge, otherwise there is no connection.

### Null-distribution of mutual information values

In order to determine the statistical significance of the mutual information values between genes we test for each pair of genes the following null hypothesis.


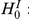
 The mutual information between gene 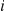 and 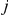 is zero.

Because we are using a nonparametric test we need to obtain the corresponding null distribution for 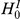 from a randomization of the data. Principally, there are several ways to perform such a randomization which conform with the formulated null hypothesis. For this reason, we perform 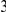 different randomizations and compare the obtained results with respect to the performance of the inference method to select the most appropriate one. Two randomization schemes (RM1 and RM2) permute the expression profiles for *each gene pair* separately. RM1 permutes *only* the sample labels and RM2 permutes the sample *and* the gene labels. In contrast, the randomization scheme RM3 permutes the sample and gene labels *for all genes* of the entire expression matrix at once.

### Mutual Information Estimators

Due to the expected nonlinearities in the data we use mutual information estimators to assess the similarity between gene profiles instead of correlation coefficients. In a previous study, we found that for normalized microarray data the distribution among individual gene pairs can strongly deviate from a normal distribution [41]. This makes it challenging to judge by theoretical considerations only which statistical estimator is most appropriate for gene expression data because most estimators were designed assuming normal data. For this reason we compare eight different estimators and investigate their influence on the performance of C3NET.

Mutual Information is frequently estimated from the marginal and joint entropy 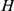 of two discretized random variables 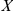 and 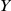 [42],
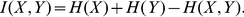
(8)


In our study, we use four MI estimators based on continuous data and four MI estimators based on discretized data. The MI estimators for discretized data are the empirical estimator [42], Miller-Madow [42], shrinkage [43] and the Schürmann-Grassberger [44] mutual information estimator. For the emipirical estimator, the entropy 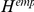 is estimated from the observed cell frequencies for each bin 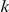 of a random variable discretized into 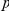 bins, i.e.,
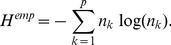
(9)With an increasing number of bins, the empirical estimator underestimates the true entropy 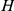 due to undersampling of the cell frequencies 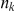. The different estimators attemp to adjust the undersampling bias by a constant factor [42], estimate cell frequencies by a shrinkage function between two models [43] or add a pseudo count from a probability distribution to the cell frequencies [44].

Mutual information can also be estimated from continuous random variables. The B-spline estimator considers the bias induced by the discretization for values falling close to the boundaries of a bin. For each bin, weights are estimated for the corresponding values from overlapping polynomial B-spline functions [45]. Hence, this method allows to map values to more than one bin.

For normal data, there is an analytical correspondence between a correlation coefficient and the mutual information [25],
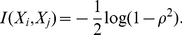
(10)In this equation, the coefficient 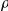 could be the Pearson correlation coefficient 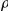, Spearman rank correlation coefficient 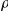 or the Kendal rank correlation coefficient 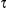.

### Yeast gene expression data

We use the *S. cerevisiae* Affymetrix ygs98 RMA normalized gene expression compendium available from the Many Microbe Microarrays Database M3D [46]. The yeast compendium dataset comprises 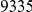 probesets and 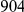 samples from experimental and observational data from anaerobic and aerobic growth conditions, gene knockout and drug perturbation experiments. We map the yeast affymetrix probeset IDs to gene symbols using the annotation of the *ygs98.db* Bioconductor package. Multiple probesets for the same gene are summarized by the median expression value. The resulting expression matrix comprises a total of 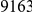 features for 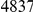 gene symbols and 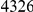 probesets that cannot be assigned to a gene symbol.

### Simulated gene expression data

We simulate a variety of different gene expression datasets for Erdös-Rényi networks [47] with an edge density of 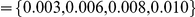. An Erdös-Rényi network is generated by starting with 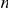 unconnected vertices. Then, between each vertex pair an edge is included with a pre-selected probability. The generated networks contain 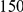 genes of which 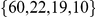 genes are unconnected. For each network, simulated gene expression datasets were created for various sample sizes of 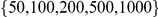 by using Syntren [48] including biological noise. We generate also simulated gene expression datasets for different subnetworks from the *E.coli* transcriptional regulatory network obtained from RegulonDB. The giant connected component (GCC) of the transcriptional regulatory network of *E.coli* consists of 1192 genes. We sample seven connected subnetworks from the GCC of sizes 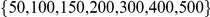. Again, using Syntren we simulate 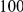 different expression datasets including biological noise with sample size 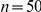 for each of these seven networks.

### Gene pair enrichment analysis (GPEA)

To test the enrichment of GO-terms in the inferred yeast BC3NET network we adopt a hypergeometric test (one-sided Fisher exact test) for edges (gene pairs) instead of genes in the following way. For 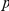 genes there is a total of 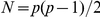 different gene pairs. If there are 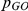 genes for a given GO-term then the total number of gene pairs is 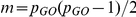. Suppose the inferred yeast BC3NET network contains 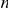 edges of which 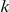 are among genes from the given GO-term, then a p-value for the enrichment of this GO-term can be calculated from a hypergeometric distribution by
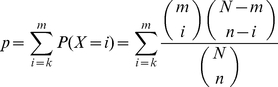
(11)Here the p-value estimates the probability to observe 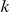 or more edges between genes from the given GO-term. For all GO:0032991 (macromolecular complex) offspring terms from Cellular Component that correspond to protein complexes, the above null hypothesis reflects the expected connection in a protein complex which is a clique (fully connected). For all other GO categories that we test, e.g., from the category Biological Process, the above is a very conservative assumption.

## Results

### Influence of the randomization and MTC

**Figure 2:**
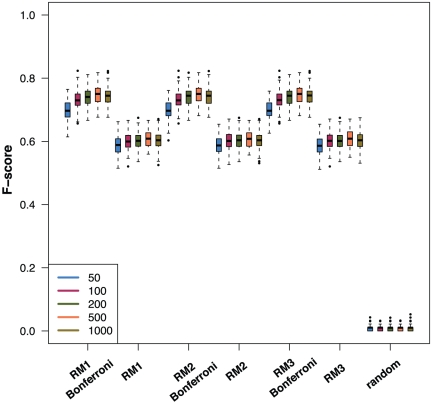
Influence of different randomization schemes (*RM1*, *RM1* and *RM3*) and the multiple hypothesis testing correction on the network inference performance, measured by the F-score. The legend shows the used sample sizes. Each randomization scheme is used with and without a Bonferroni correction. The boxplots labeled ‘random’ correspond to randomly permuted data to get an impression for random F-scores.

The influence of the randomization scheme on the performance of BC3NET is shown in Fig. 2. Here we use simulated data from a Erdös-Rényi network consisting of 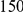 genes, of which 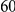 are unconnected. The figure shows results for RM1-RM3 with and without MTC for five different sample sizes, shown in the legend of the figure. As one can see, all three randomization schemes with a Bonferroni correction perform similarly good. Also RM1-RM3 without MTC perform similarly, however, significantly worse indicating the importance to correct for multiple hypotheses testing. Due to the fact that RM3 is from a computational point of view more efficient than RM1 or RM2 we use this randomization scheme for our following investigations.

Fig. 2 includes also the F-scores obtained from the randomization of the expression data itself (right-hand side) to obtain baseline values for a comparison with the results from RM1-RM3. This is interesting because, e.g., in contrast to the AU-ROC [49], the F-score for data containing only noise is not 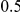 as for the AU-ROC. From this perspective, one can see that even the results without MTC are significantly better than expected by chance.

### Influence of the mutual information estimator

**Figure 3:**
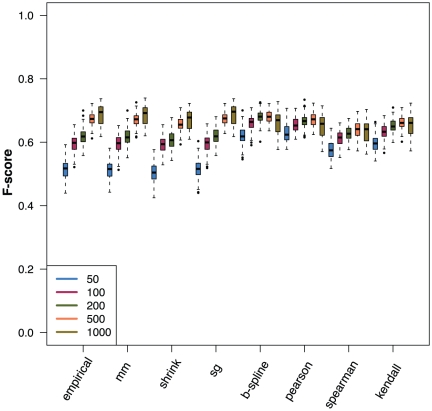
Influence of the statistical mutual information estimators (x-axis) on the network inference performance, measured by the F-score. The legend shows the used sample sizes. Gene expression data were simulated for an Erdös-Rényi network with 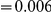.

To study the influence of the statistical estimators of the mutual information values, we use simulated data for several different network topologies. Fig. 3 shows results for eight different estimators and different sample sizes for a Erdös-Rényi network with an edge density of 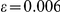. The three continuous estimators, Pearson, Spearman and Kendall as well as B-spline, perform better for smaller sample sizes. For large sample sizes the empirical, Miller-Madow, shrinkage and Schürmann-Grassberger perform slightly better. We want to note that for different parameters of the Erdös-Rényi network and different network types we obtain qualitatively similar results (not shown). Considering the size of the studied networks we used for our analysis, which contain 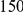 genes, sample sizes up to 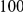 lead to a realistic ratio of 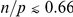 which one can also find for real microarray data. Larger ratios are currently and the near future hard to achieve. For this reason, we assess the results for smaller sample sizes as more important, due to their increased relevance for practical applications. Based on these results we use for the following studies the B-spline estimator.

### Comparative analysis of BC3NET

#### Computational complexity

In [9] the computational complexity of C3NET has been estimated as 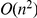, where 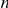 corresponds to the number of genes. For BC3NET this means that its computational complexity is 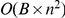. Here 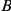 is the number of bootstraps. In order to provide a practical impression for the meaning of these numbers, we compare the computational complexity between the ARACNe bootstrap network approach, described in [33], and BC3NET. We performed an analysis for a gene expression data set with 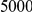 genes and 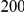 samples. The ARACNe algorithm needed 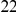 hours for a single run that means to analysis one bootstrap data set. This results in a total time of 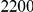 hours (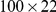 hours) for 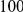 bootstraps, which are about 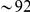 days. In contrast, the BC3NET algorithm completed this task in only 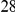 minutes for all 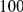 bootstraps.

#### Comparative analysis using simulated data

**Figure 4:**
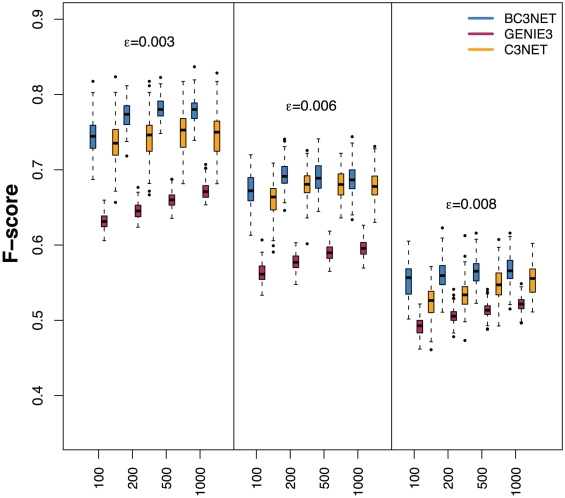
Comparative analysis of BC3NET, GENIE3 and C3NET for Erdös-Rényi networks with edge density 
 **.** The x-axis shows the sample size 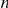.

In order to gain insight into the quality of BC3NET we study it comparatively by contrasting its performance with GENIE3 and C3NET. In Fig. 4 we show results for three different Erdös-Rényi networks each with 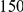 genes, of which 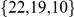 genes are unconnected. The edge density of these networks is 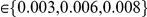. We use these edge densities because regulatory networks are known to be sparsely connected [50]. The F-score distributions for all studied conditions are larger for BC3NET. We repeated the above simulations for subnetworks from the transcriptional regulatory network of *E. coli* and obtained qualitatively similar results. This demonstrates the robustness of the results with respect to different network types and network parameters.

**Figure 5:**
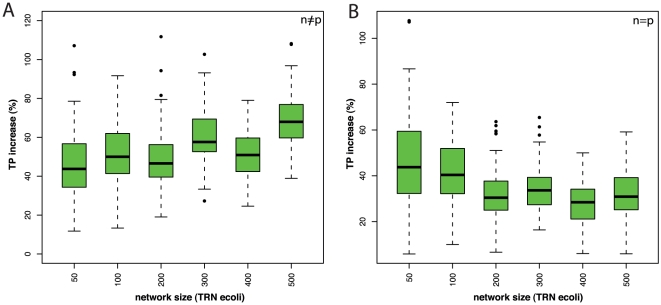
Gain in the number of true positive edges in BC3NET compared with C3NET. The x-axis shows the size (number of genes 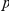) of the used subnetwork of *E.coli*. A: Influence of network size on TP gain with constant sample size 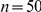 (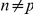). B: Influence of network size on TP gain with sample size 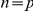.

To emphasize the actual gain in the number of true positive edges with respect to C3NET, on which BC3NET is based, we present in Fig. 5 the percentage of the increase of inferred true positive edges for various network sizes ranging from 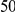 to 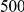 genes for subnetworks from *E. coli*. For the results shown in the left figure, we use a fixed sample size of 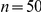 and for the right figure the sample size equals the number of genes, i.e., 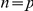. For a fixed sample size (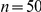) the BC3NET networks show an increase of true positives edges 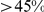, with a more prominent increase for the larger networks. Quantitatively, this observation is confirmed by a linear regression which gives a none vanishing positive slope of 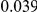 and an intercept of 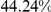. Both parameters are highly significant with p-values 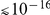. For the datasets with variable sample sizes (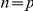) the percentage of inferred true positive edges remains constant with an increasing network size and is around 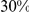. We want to note that the results for 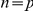 assess the asymptotic behavior of BC3NET because the number of samples 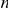 increases linearly with the number of genes 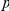. That means, asymptotically, the gain of BC3NET over C3NET is expected to be 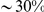. On the other hand, for real data for which 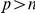 holds, the expected gain is much larger, as one can see from the left figure, reaching 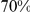.

### Analysis of the regulatory network of yeast

Using BC3NET, we infer a regulatory network for a large-scale gene expression dataset of *Saccharomyces cerevisiae*. Due to the fact that for *Saccharomyces cerevisiae* no gold standard reference network is available to assess the quality of the inferred GRN we evaluate the resulting network by using functional gene annotations and experimentally validated protein interactions.

The yeast network inferred by BC3NET is a connected network that contains 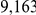 genes and 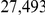 edges with an edge density of 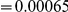. The degree distribution of this network follows a power-law distribution, 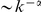, with an exponent of 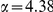. We tested a total of 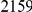 GO-terms from the category Biological Process, whereas each GO-term contains less than 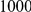 annotated genes. From these, 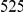 (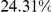) test significant using a Bonferroni procedure indicating an enrichment of gene pairs for the corresponding GO-terms. The strongest enrichment of gene pairs we find in our analysis are for ribosome biogenesis, ncRNA and rRNA processing, mitochondirial organization, metabolic and catabolic processes and cell cycle. See Table 1 for an overview of the top 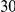 results.

**Table 1:** Top 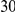 GO-terms for a GPEA for the category Biological Process.

GOID	Term	Genes	Edges	Exp	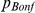
GO:0042254	ribosome biogenesis	349	546	66	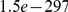
GO:0022613	ribonucleoprotein complex biogenesis	398	581	86	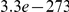
GO:0034470	ncRNA processing	332	472	60	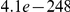
GO:0006364	rRNA processing	237	355	31	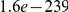
GO:0016072	rRNA metabolic process	246	364	33	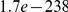
GO:0034660	ncRNA metabolic process	386	519	81	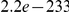
GO:0006412	translation	699	822	267	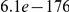
GO:0006396	RNA processing	506	581	140	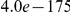
GO:0007005	mitochondrion organization	282	330	43	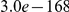
GO:0032543	mitochondrial translation	100	159	5	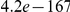
GO:0044085	cellular component biogenesis	841	905	386	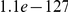
GO:0044281	small molecule metabolic process	890	847	432	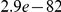
GO:0044257	cellular protein catabolic process	347	271	66	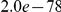
GO:0030163	protein catabolic process	369	288	74	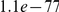
GO:0006082	organic acid metabolic process	388	303	82	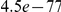
GO:0044248	cellular catabolic process	720	612	283	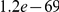
GO:0006519	cellular amino acid and derivative metabolic process	296	216	48	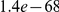
GO:0009056	catabolic process	810	709	358	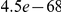
GO:0019752	carboxylic acid metabolic process	370	271	75	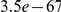
GO:0043436	oxoacid metabolic process	370	271	75	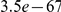

All terms contain 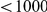 and 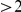 genes. ‘Exp’ denotes the expected number of edges for a GO-term. A total of 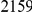 terms were tested of which 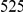 (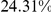) tested significant.

One of the most reliable to detect (biochemical) interaction types that can be experimentally tested and that correspond to *causal* interactions, are protein-protein interactions from protein complexes. The reason therefore is that protein-protein interactions establish a direct connection between the proteins by forming physical bonds. Therefore we study the extend of protein complexes, as defined in the GO database [51], that are present in the yeast BC3NET network. We perform GPEA for 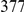 GO-terms, which correspond to different protein complexes. From these we identify 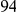 protein complex terms with significantly enriched gene-pairs. The top 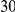 GO-terms of protein complexes we find are listed in Table 2. Some of the largest protein complexes detected in the BC3NET network are ribonucleoprotein complexes (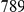 edges) including the cytosolic ribosome (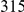 edges) and mitochondrial ribosome (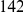 edges). Further protein complexes present in the yeast BC3NET network are the proteasome complex (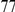 edges), proton-transporting ATP synthase complex (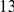 edges) and DNA-directed RNA polymerase complex (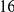 edges).

**Table 2:** Top 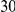 GO-terms for a GPEA for protein complexes.

GOID	Term	Genes	Edges	Exp	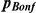
GO:0033279	ribosomal subunit	210	442	24	0
GO:0022626	cytosolic ribosome	151	315	12	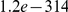
GO:0005840	ribosome	291	485	46	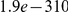
GO:0030529	ribonucleoprotein complex	568	789	176	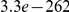
GO:0000313	organellar ribosome	78	142	3	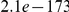
GO:0005761	mitochondrial ribosome	78	142	3	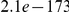
GO:0015934	large ribosomal subunit	124	154	8	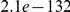
GO:0030684	preribosome	130	155	9	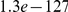
GO:0000502	proteasome complex	49	77	1	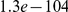
GO:0022625	cytosolic large ribosomal subunit	82	96	4	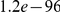
GO:0000315	organellar large ribosomal subunit	42	58	1	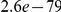
GO:0005762	mitochondrial large ribosomal subunit	42	58	1	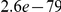
GO:0031597	cytosolic proteasome complex	30	45	0	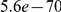
GO:0034515	proteasome storage granule	30	45	0	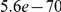
GO:0015935	small ribosomal subunit	86	69	4	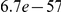
GO:0022627	cytosolic small ribosomal subunit	54	48	2	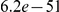
GO:0030686	90S preribosome	80	55	3	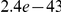
GO:0005838	proteasome regulatory particle	24	27	0	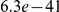
GO:0022624	proteasome accessory complex	24	27	0	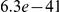
GO:0005839	proteasome core complex	15	21	0	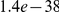

All terms contain more than 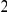 genes. A total of 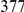 different terms were tested of which 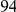 protein complexes (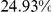) were significant.

Finally, we study experimentally evaluated protein-protein interactions extracted from the BioGrid database (release 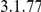) [52] and compare them with our yeast BC3NET network. First, we find that the yeast PPI network from BioGrid and our yeast BC3NET network have 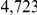 genes in common. Further, we find a total of 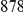 BioGrid interactions among 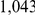 genes that are present in the yeast BC3NET network. These interactions are distributed over a total of 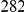 separate network components, each consisting of 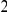 or more genes. Among these, we find 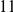 network components with a significant component size, where the largest significant component includes 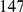 genes and the smallest significant component includes 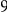 genes. Significance was identified from gene-label randomized data generating a null distribution for the size of connected network components of the 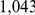 genes. The resulting p-values were Bonferroni corrected. For each BioGRID component that is nested in the yeast BC3NET network, we conduct a GO enrichment analysis. From this analysis we use the GO-term with the highest enrichment value to annotate the individual network components, see Table 3.

**Table 3:** Shown are 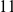 significant BC3NET network components nested in the BioGrid PPI yeast network.

Component	Genes	Edges	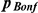	GO
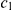	147	210	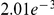	ribosome biogenesis ( 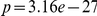 )
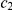	49	50	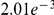	protein amino acid glycosylation ( 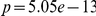 )
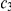	41	69	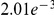	ubiquitin-dependent protein catabolic process ( 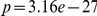 )
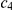	22	21	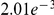	actin cytoskeleton organization ( 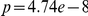 )
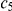	22	25	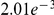	DNA replication ( 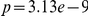 )
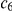	19	19	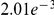	mitochondrial translation ( 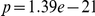 )
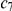	15	17	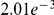	ergosterol biosynthetic process ( 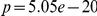 )
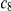	10	10	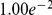	cytokinesis ( 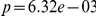 )
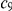	10	9	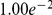	DNA replication initiation ( 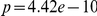 )
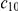	10	12	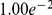	response to pheromone ( 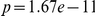 )
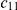	9	9	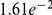	microtubule-based process ( 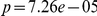 )

Shown are the number of genes and concordant edges for each BC3NET network component. The p-values were adjusted using a Bonferroni procedure. We annotated these network components by using the most enriched GO term from the category Biological Process.

**Figure 6:**
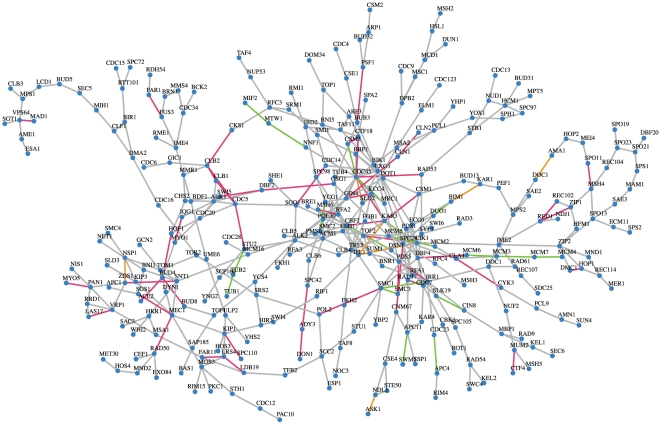
The largest network component of the yeast BC3NET network nested in *cell cycle* (GO category Biological Process: GO:0007049). The network component comprises 304 genes and 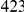 edges (GPEA *cell cycle* 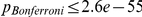). The 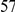 violet edges correspond to interactions present in BioGrid, 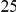 green edges correspond to protein-protein interactions in protein complexes (GO) and the 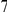 orange edges are present in both databases.

One of the most extensively studied biological processes in *Saccharomyces cerevisiae* is the cell cycle. For cell cycle the GPEA gives a gene-pair enrichment p-value of 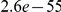, see Table 1. In Fig. 6 we show the largest network component of the cell cycle inferred by BC3NET that includes 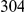 genes and 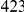 edges. From this network, 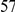 edges are confirmed in BioGrid (violet edges), 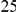 edges are from protein complex units (GO) (green edges) and 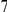 edges are present in both databases (orange edges).

## Discussion

From the analysis of BC3NET for the gene expression data set from *S. cerevisiae*, we find in addition to a significant enrichment of over 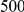 GO-terms in the category Biological Process, the significance of 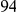 GO-terms in Cellular Component for protein complexes. The largest complexes we identified are the ribosome (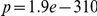) and proteasome protein complex (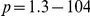). There are two main reasons why edges of these protein complexes are highly abundant in the yeast BC3NET network. First, the ribosome and proteasome protein complexes are well annotated because they have been extensively studied in yeast [53]. Second, the ribosome and proteasome protein complex are mainly regulated on the gene expression level and, where observed, having highly dependent gene expression patterns [53]. Therefore, it is plausible that GRN inference methods can also pick-up signals from physical interactions between protein subunits of protein complexes.

We want to note that we are not the first to recognize that gene expression data contain information about protein-protein interactions. For example, [53], [54] provide evidence that proteins from the same complex show a significant coexpression of their corresponding genes. Also in [55] it is mentioned that inferred interactions from gene expression data ‘may represent an expanded class of interactions’ [55]. However, when it comes to the experimental assessment of the inferred networks, usually, only interactions related to the transcriptional regulation are studied, e.g., with ChIP-chip experiments [11], [16]. To our knowledge we are the first to provide a large-scale analysis of an inferred GRN from gene expression data with respect to the presence of protein-protein interactions.

BC3NET is an ensemble method that uses as base network inference algorithm C3NET [9], [56]. As for other ensemble methods based on bagging, e.g., random forests, the interpretability and characteristics of the base method does usually not translate to the resulting ensemble method [28], [32]. In our case this means that the inferred network can actually have more than 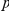 edges, despite the fact that networks inferred by C3NET can not. However, in our case this is a desirable property because it improves BC3NET leading ultimately to a richer connectivity structure of the inferred network. Specifically, our numerical results demonstrate that BC3NET gains in average more than 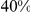 true positive edges compared to C3NET (see Fig. 5). Another more general advantage of an ensemble approach is that it is straight forward to use on a computer cluster because a parallelization is naturally given by the base inference methods. Given the increasing availability of computer clusters this appears to be a conceptual advantage over none ensemble methods, likely to gain even more importance in the future. In this paper we pursued a conservative approach by using a Bonferroni procedure for MTC to demonstrate that even in this setting our method is capable of inferring many significant interactions that can be confirmed biologically. However, there is certainly potential to use more adopted MTC procedures that are less conservative. For example, procedures controlling the *false discovery rate* (FDR) could be investigated [39], [57].

Further, we want to note that despite the fact that the network inference method C3NET is no Bayesian method [58], [59], BC3NET is. The reason for this is that it is known for the bootstrap distribution of a parameter to correspond approximately to the Bayesian posterior distribution for a noninformative prior, and the bagged estimate thereof is the approximate mean of the Bayesian posterior [60]. Hence, BC3NET can be considered as a Bayesian method with noninformative priors for the connectivity structure among the genes. Given the problem to define informative priors for a Bayesian approach in a genomics context, either because not enough reliable information about a specific organism is available or because it is difficult to select this information in an uncontroversial manner, a noninformative prior is in the current state of genomics research still a prevalent choice. From a theoretical point of view, a bootstrap implementation is easier to accomplish than the corresponding (full) Bayesian method. Hence, our approach is more elementary [60]. Employing a similar argument as above, one can also see that BC3NET performs a model averaging of the individual networks inferred by C3NET.

From a conceptual point of view, one may wonder if an inferred GRN using BC3NET corresponds to a causal or an association network [19], [61]. Here, by *causal* we denote an edge that corresponds to a *direct* interaction between gene products, e.g., the binding of a transcription factor to the promoter region on the DNA for regulating the expression of this genes. The quantitative evaluation of our simulated data, provide actually a quantification of the *causal content* of the inferred networks in the form of F-scores. It is clear that due to the statistical nature of the data, any inference is accompanied by a certain amount of uncertainty leading to an inferred GRN that contains false positive as well as false negative edges. However, as demonstrated by our numerical analysis, BC3NET is an important improvement toward the inference of causal gene regulatory networks.

Despite the fact that the presented inference method BC3NET was introduced by using gene expression data from DNA microarray experiments, it can also be used in connection with data from RNA-seq experiments. Given the rapidly increasing importance of this new technology we expect that within the next few years datasets with sufficient large sample size are available to infer GRN.
